# Effect of upper torso inclination in Fowler’s position on autonomic cardiovascular regulation

**DOI:** 10.1007/s12576-013-0273-8

**Published:** 2013-07-02

**Authors:** Satoshi Kubota, Yutaka Endo, Mitsue Kubota

**Affiliations:** School of Nursing and Rehabilitation Sciences at Odawara, International University of Health and Welfare, 1-2-25 Shiroyama, Odawara, Kanagawa 250-8588 Japan

**Keywords:** Fowler’s position, Autonomic cardiovascular regulation, Respiratory sinus arrhythmia, Cardiovagal baroreflex sensitivity, Transfer function analysis

## Abstract

The present study investigates autonomic cardiovascular regulation during postural changes while in Fowler’s position. Respiratory sinus arrhythmia (RSA) and sequence baroreflex sensitivity (sBRS) were measured in 12 healthy individuals in three positions (Experiment 1). We also measured RSA, sBRS, tidal volume (TV), lung volume spectrum (LV spectrum), and transfer gain and phase between lung volume and RR interval (RSA-TF, RSATF-phase) in 11 healthy individuals in two positions (Experiment 2). All participants maintained respiratory frequency at 15 breaths/min. The three positions in Experiment 1 were 30°, 45°, and 60° of upper torso inclination with a lower torso inclination of 30° throughout all evaluations. The two positions in Experiment 2 were 30° and 60° of upper torso backrest inclination with a lower torso inclination of 30° throughout all evaluations. The results of Experiment 1 showed significantly higher RSA and sBRS at 60° and 45° than at 30°, whereas RR interval (RRI), systolic blood pressure (SBP), and diastolic blood pressure (DBP) did not differ significantly under any condition. The results of Experiment 2 showed that RSA, RSA-TF, sBRS, TV, and LV spectrum were significantly higher at 60° than at 30°, and that RRI, SBP, DBP, and the RSATF phase did not significantly differ under any condition. These findings suggested that slight flexion of the upper torso in Fowler’s position activates respiratory function and increases the contribution of vagal nerve activity to the cardiovascular system in young participants under conditions of a fixed respiratory rate.

## Introduction

Studies on autonomic regulation of the cardiovascular system in humans with the head tilted up (HUT) and down (HDT) have found that posture influences the autonomic nervous system. Generally, standing or tilting the head upwards increases sympathetic nerve activity and decreases vagus nerve activity, vagal modulation, and vagal baroreflex sensitivity. Conversely, sympathetic nerve activity decreases in the supine position, whereas vagus nerve activity, vagal modulation, and vagal baroreflex sensitivity increase [1–6]. These facts were determined by analyzing heart periods, blood pressure fluctuations, and muscle sympathetic nerve activity. The normal response to the above is the autonomic regulation of cardiovascular function according to changes in the distribution of blood volume. Moreover, tidal volume (TV) is significantly increased in tilted, compared with the supine position via the activation of vestibular reflex and lung–thoracic wall afferents [7]. In addition, an increase in TV might enhance the vagal modulation and the cardiovagal baroreflex [8–10].

The Fowler’s or semi-seated position as well as the standing and supine positions are often clinically applied. The Fowler’s position is achieved by inclining the backrest of a bed upwards from the supine position with flexed or straight knees [11]. It is frequently used instead of the supine position to monitor hemodynamics and facilitate breathing and daily activities such as eating or conversation in frail patients [11–14]. Fowler’s position is clinically applied most frequently at inclinations between 30° and 60° [11, 14]. Some studies have described a relationship between the angle of Fowler’s position and the accuracy of hemodynamic measurements among patients in intensive care units [15–17], and Driscoll et al. [17] reported that cardiac output is decreased in Fowler’s, compared with the supine position among patients in intensive care. A study of young healthy individuals has shown that blood pressure values in Fowler’s position are intermediate between the seated and supine positions [18]. Moreover, a cross-sectional study of hypertensive patients found the same tendency [19]. However, to our knowledge, the effects of slight postural differences in Fowler’s position on cardiovascular regulation and hemodynamics have not been investigated. The physiological influences of various positions should be understood to improve patient care in the clinical setting.

We speculated that the distribution of blood volume does not predominantly change because most body segment positions remain unaltered regardless of upper torso flexion in Fowler’s position, whereas TV increases with upper torso flexion and consequently enhances cardiovagal regulation. We tested this hypothesis by investigating the effects on heart period fluctuation [respiratory sinus arrhythmia (RSA)] and baroreflex sensitivity with and without upper torso flexion in Fowler’s position on the autonomic regulation of cardiovascular function.

## Methods

### Participants

Experiment 1. We assessed RSA and baroreflex sensitivity in 12 healthy adult volunteers (mean age ± SEM, 20.3 ± 0.3 years; range 19–22 years; weight 54.3 ± 2.0 kg; height 164.3 ± 2.0 cm; male and female, *n* = 6 each).

Experiment 2. We assessed the influence of tidal volume on RSA in 11 healthy male adult volunteers (mean age ± SEM, 20.0 ± 0.5 years; range 18–22 years; weight 59.5 ± 1.9 kg; height 170.8 ± 1.5 cm).

 Most of the participants were university students, and all were free of chronic or acute cardiovascular, respiratory, or other chronic diseases. Experiments involving females proceeded within the follicular phase of the menstrual cycle. Beverages containing caffeine or alcohol were not consumed for 24 h before starting the study. All participants refrained from eating and drinking after 2200 h on the evening before experiments that proceeded in the morning or consumed a light breakfast before experiments that proceeded in the afternoon. Male participants wore only a pair of shorts and female participants wore a sleeveless top and shorts.

All experiments proceeded between 1030 and 1300 h. Cardiovascular function and ventilation were monitored in seated participants. The study conformed to the published recommendations of the Ethics Commission of the International University of Health and Welfare and all volunteers provided written informed consent to participate.

### Procedure

The participants rested in a thermoneutral room at 28 °C for 15 min and were then prepared to undergo electrocardiography (ECG) and continuous measurements of arterial blood pressure and respiratory wave forms. The participants remained seated in each condition for 2 min and then recordings were collected for 10 min under each condition in all experiments. Changes in respiratory frequency and tidal volume can affect RR interval fluctuations and cardiac vagal outflow [20–25]. Respiratory frequency was controlled at 15 breaths/min (0.25 Hz) using a metronome. The influence of tidal volume on RR interval fluctuations in Experiment 2 was examined under two of the three conditions described for Experiment 1. We analyzed data collected during the last 5 min of 10-min recordings.

**Fig. 1:**
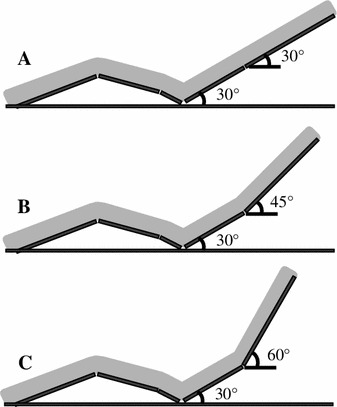
Seats used for each condition.**a** Upper and lower torso inclined at 30°. **b** Upper and lower torso inclined at 45° and 30°, respectively. **c** Upper and lower torso inclined at 60° and 30°, respectively. (Experiment 1: 30°, 45°, and 60°; Experiment 2: 30° and 60°)

The seat backrest in Experiment 1 provided upper torso inclination angles of 30°, 45° and 60° and a lower torso angle of 30° (Fig. 1). All seated conditions were the same and allowed slight hip and knee joint flexion. Experiment 1 was repeated on different days and all three conditions were examined at random.

The seats for Experiment 2 provided upper torso inclination angles of 30° and 60° (Fig. 1) with the same lower torso inclination angle and leg support, as described above. Experiment 2 was repeated on the same day in random order of conditions.

### Instrumentation

Data from a LEG-1000 electrocardiographic lead II (Nihon Kohden, Tokyo, Japan), continuous arterial blood pressure (BP-608EV; Omron Colin, Tokyo, Japan) and respiratory wave forms measured using a nose-tip thermistor (LEG-1000; Nihon Kohden) were recorded on a personal computer through a PCMCIA-GPIB 186736C-01 analog-to-digital converter (National Instruments, Austin, TX, USA) at a sampling rate of 1 kHz throughout Experiment 1. Continuous arterial blood pressure was measured at the radial artery by tonometry using a noninvasive arterial blood pressure monitor at the heart level. Continuous arterial blood pressure was calibrated using oscillometric sphygmomanometry to measure intermittent cuff blood pressure.

Data from an ECG 100C electrocardiographic lead II (BIOPAC Systems, Goleta, CA, USA), continuous arterial blood pressure measured as described above and lung volume (LV) measured using a TSD117 air flow transducer (BIOPAC Systems) were recorded on a personal computer using the MP150 data acquisition system (BIOPAC Systems) at a sampling rate of 1 kHz throughout Experiment 2. We measured LV using an air flow transducer attached to a face mask.

### Data analysis

#### Analysis of hemodynamics

We detected RR intervals (RRI) from ECG data in both experiments. Systolic (SBP) and diastolic (DBP) blood pressure was determined from continuous arterial blood pressure. We calculated 5-min mean values for RRI, SBP, and DBP. Tidal volumes (TV) were detected from the LV in Experiment 2.

#### Spectral analysis and transfer function analysis

The RRI and LV time series were linearly interpolated and resampled at 2 Hz and then subdivided into 256-point segments with a 50 % overlap. First Fourier Transform was applied to the power spectrum of the RRI data in both experiments. We then measured RSA (0.15–0.4 Hz), which is an oscillation of heart rate in synchrony with respiration. The RSA is the high-frequency component of the RRI power spectrum data, which reflects the vagal modulation of heart rate and serves as an index of cardiac parasympathetic control [26–29].

We applied gain between lung volume and RRI for transfer function analysis in ms/mL of change in lung volume during Experiment 2 as described [29–31]. The frequency at which transfer gain was assessed ranged from 0.15 to 0.4 Hz, reflecting RSA. Coherence between lung volume and RRI at this frequency had to be at least 0.5 for accurate evaluation of the transfer gain because lower coherence indicates that a factor(s) other than lung volume fluctuation is responsible for RRI fluctuation. Thus, we also applied the phase (RSATF-phase: 0.15–0.4 Hz) for transfer function analysis of the degree and power spectrum of lung volume (LV spectrum: 0.15–0.4 Hz) in Experiment 2. We used DIAdem time series analysis software (National Instruments) for spectral and transfer function analyses.

#### Sequence baroreflex sensitivity

The beat-to-beat time series of RRI and SBP were scanned to identify sequences when RRI and SBP increased or decreased over ≥3 consecutive beats. In addition, the minimum change threshold was 1 mmHg for SBP and 4 ms for RRI. Linear regression with correlation coefficients for sequences >0.85 was analyzed. The mean values of the individual slopes of all SBP-RRI sequences were calculated as sequence baroreflex sensitivity (sBRS). The sBRS reflects vagally mediated cardiac baroreflex responses [32, 33], because these baroreflex sequences (increases or decreases during ≥3 consecutive beats of SBP-RRI) predominantly reflect the vagal branch of the baroreflex [34]. Sequence baroreflex sensitivity was analyzed using Nevrokard BRS software (Nevrokard, Izola, Slovenia).

### Statistical analysis

Experiment 1. We determined the effects of thoracic inclinations of 30°, 45° and 60° upon physiological variables using a repeated measures multivariate analysis of variance (MANOVA) with Pillai’s trace statistic. Differences between two conditions were evaluated using a paired *t* test with Shaffer’s multiple comparison procedure [35] when a main group effect was significant.

Experiment 2. Differences between 30° and 60° were evaluated using a paired *t* test. Statistical significance was established at *p* < 0.05. All values are described as mean ± SEM. Data were statistically analyzed using R for Windows v.2.13 (http://www.r-project.org) and the car 2.0–11 package for MANOVA.

## Results

### Experiment 1

**Fig. 2:**
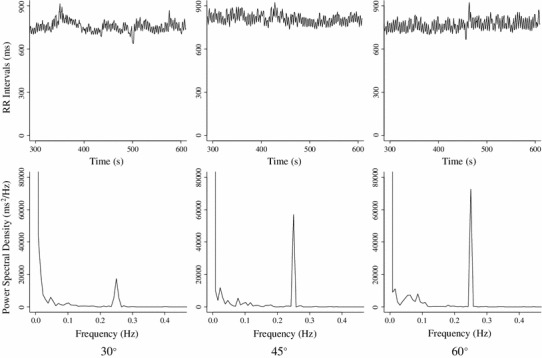
Typical data of time series of RR interval (*top panels*) and power spectral density of RR interval (*lower panels*) in one participant under all conditions (*left*, *middle* and *right panels*: 30°, 45° and 60°, respectively). Peak of power spectral density around 0.25 Hz increased with increased upper torso angle (*bottom panels*)

**Fig. 3:**
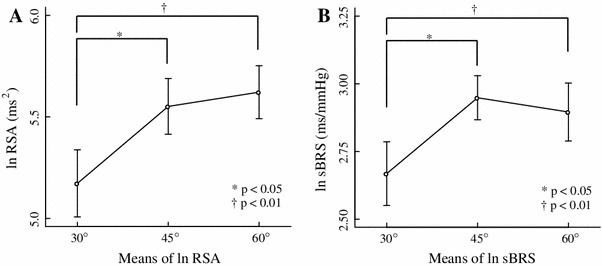
Results of inclination at 30°, 45°, and 60° (Experiment 1). Means (±SEM) of logarithm-transformed respiratory sinus arrhythmia (ln RSA) (**a**) and logarithm-transformed sequence baroreflex sensitivity (ln sBRS) (**b**). *p < 0.05; ^†^ *p* < 0.01

Table 1 shows the results of Experiment 1 and Fig. 2 shows data typical of a time series and the power spectral density of RRI in an individual under all conditions tested. About 0.25 Hz corresponds to the respiratory frequency of the power spectral density of RRI. The peak of power spectral density around 0.25 Hz increased with an increase in the upper torso angle. Figure 3 shows the logarithm (ln) RSA and ln sBRS under all three conditions. Both ln RSA and ln sBRS exerted significant major effects (ln RSA, *F*_2,10_ = 9.65, *p* < 0.01; ln sBRS, *F*_2,10_ = 7.29, *p* < 0.05; MANOVA) at all three angles. Multiple comparison tests showed that RSA (30°, 45° and 60°: 5.17 ± 0.17, 5.55 ± 0.14, and 5.62 ± 0.13 ms^2^, respectively) and ln sBRS (30°, 45° and 60°: 2.62 ± 0.12, 2.95 ± 0.08, and 2.90 ± 0.11 ms/mmHg, respectively) were significantly higher at 60° (*p* < 0.01) and 45° (*p* < 0.05) than at 30°. Neither RSA nor sBRS significantly differed between these two conditions.

**Table 1:** Hemodynamics, RSA and sBRS associated with three angles of inclination in Experiment 1

	30°	45°	60°	
	Mean ± SEM	Mean ± SEM	Mean ± SEM	
RRI (ms)	921.80 ± 33.47	946.23 ± 25.00	941.75 ± 31.78	ns
SBP (mmHg)	110.80 ± 3.23	108.75 ± 2.41	112.93 ± 3.20	ns
DBP (mmHg)	63.07 ± 1.86	61.08 ± 1.96	64.33 ± 1.81	ns
ln RSA (ms^2^)	5.17 ± 0.17	5.55 ± 0.14	5.62 ± 0.13	* ^†^
ln sBRS (ms/mmHg)	2.67 ± 0.12	2.95 ± 0.08	2.90 ± 0.11	* ^†^

Values are shown as mean ± SEM

*ns* no significant difference among all conditions

* p < 0.05 between 30° and 45°

^†^ *p* < 0.01 between 30° and 60°

The effects of RRI, SBP, and DBP did not significantly differ (RRI, *F*_2,10_ = 0.66, *p* > 0.05; SBP, *F*_2,10_ = 4.02, *p* > 0.05; DBP, *F*_2,10_ = 3.48, *p* > 0.05; all MANOVA) among the three angles.

The tendencies of all data were the same for males and females.

### Experiment 2

**Fig. 4:**
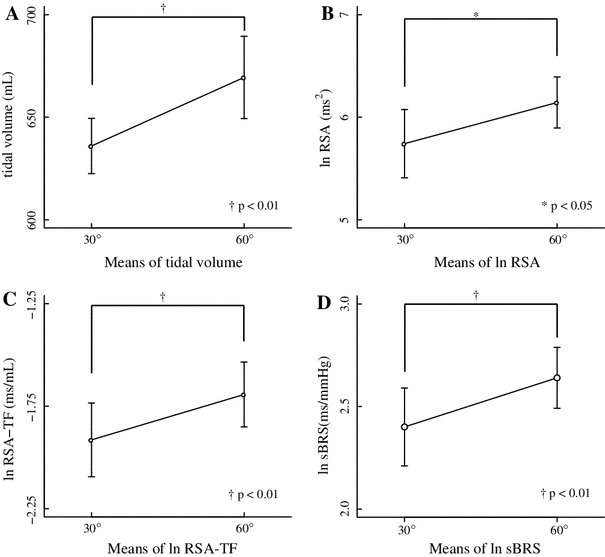
Results of each inclination at 30° and 60° (Experiment 2). Means (±SEM) of tidal volume (**a**), ln RSA (**b**), logarithm-transformed transfer gain between respiratory sinus arrhythmia and tidal volume (ln RSA-TF) (**c**), and ln sBRS (**d**). *p < 0.05; ^†^ *p* < 0.01

Table 2 shows the results of Experiment 2 and Fig. 4 shows ln RSA, ln RSA transfer gain (RSA-TF), and ln sBRS. The results of the paired *t* test showed that ln RSA (30° and 60°: 5.74 ± 0.33 and 6.14 ± 0.25 ms^2^, respectively, *p* < 0.01), ln RSA-TF (30° and 60°: −1.89 ± 0.17 and −1.66 ± 0.16 ms/mL, respectively, *p* < 0.01), ln sBRS (30° and 60°: 2.40 ± 0.19 and 2.64 ± 0.15 ms/mmHg, respectively, *p* < 0.01), TV (30° and 60°: 635.98 ± 13.48 and 669.41 ± 20.05 mL, respectively, *p* < 0.01) and ln LV spectrum (30° and 60°: 9.65 ± 0.07 and 9.76 ± 0.07 mL^2^, respectively, *p* < 0.05) were significantly higher at 60° than at 30°. None of RRI, SBP, and DBP significantly (*p* > 0.05) differed between the two conditions. The RSATF-phase (30° and 60°: −84.55 ± 9.78 and −97.16 ± 3.53°, respectively, *p* > 0.05) did not differ significantly and was negative between the two conditions in all participants, indicating that respiration preceded RRI between 30° and 60°.

**Table 2:** Hemodynamics, RSA, RSA-TF and sBRS associated with two angles of inclination in Experiment 2

	30°	60°	
	Mean ± SEM	Mean ± SEM	
RRI (ms)	902.08 ± 29.57	910.51 ± 31.43	ns
SBP (mmHg)	118.42 ± 3.91	119.19 ± 4.12	ns
DBP (mmHg)	59.55 ± 3.27	59.77 ± 2.37	ns
TV (mL)	635.98 ± 13.48	669.41 ± 20.05	**
ln RSA (ms^2^)	5.74 ± 0.33	6.14 ± 0.25	*
ln LVspectrum (mL^2^)	9.65 ± 0.07	9.76 ± 0.07	*
ln RSA-TF (ms/mL)	−1.89 ± 0.17	−1.66 ± 0.16	**
RSATF-Phase (degree)	−84.55 ± 9.78	−97.16 ± 3.53	ns
ln sBRS (ms/mmHg)	2.40 ± 0.19	2.64 ± 0.15	**

Values are mean ± SEM

*ns* no significant difference between 30° and 60°

* p < 0.05 between 30° and 60°

** p < 0.01 between 30° and 60°

## Discussion

Slight flexion of the upper torso in Fowler’s position resulted in increased RSA, RSA-TF, sBRS and TV in healthy young adults (Table 1; Fig. 3). These findings suggested that the increase in tidal volume caused by slight flexion of the upper torso while in Fowler’s position enhances the cardiovagal baroreflex and generates RSA. A slight difference in torso posture in Fowler’s position might affect cardiovascular and respiratory regulation.

### Factors involved in the increase in tidal volume caused by upper torso flexion

Torso flexion might change respiratory patterns, since TV was increased by upper torso flexion (Table 2; Fig. 4). These results are similar to those of Yoshizaki et al. [7], who found that TV significantly increased when the head is tilted upwards (75°) compared with the supine position, and that respiratory frequency did not significant differ between the two positions in males. They described that afferent signals of the vestibular nerve in the upright position increase respiratory motor output, to which the activated vestibular reflexes contribute [36, 37]. They also described that lung–thoracic wall afferents activated by tilting might augment ventilation, possibly through decreased alveolar ventilation while supine as a result of C-fibers being activated by increased pulmonary blood volume [38].

The flexed upper torso in Fowler’s position (60°) might have stimulated the vestibular system and activated the lung–thoracic wall afferents in the present study, which might have augmented ventilation as described by Yoshizaki et al. [7].

Furthermore, the amplitude of the LV power spectrum was about 0.25 at a fixed respiratory rate of 15 breaths/min (0.25 Hz), and this reflects the volume of change in the LV which represents TV. Thus, the increase in LV spectrum under upper torso flexion might reflect increased TV.

### Effects of tidal volume on respiratory sinus arrhythmia and baroreflex

The RSA is dependent on respiratory frequency and depth [39, 40], and slow and deep breathing enhances cardiovagal baroreflex sensitivity [8–10]. Although all experiments in the present study proceeded under a fixed respiratory rate of 0.25 Hz, TV was not controlled. The RSA and sBRS were enhanced at 45° and 60°, and TV was also enhanced at 60°. We also found that RSA-TF was enhanced at 60° compared with 30°, indicating that a small change in lung volume resulted in a large change in RRI, and that RSATF-phases were negative at 60° and 30°, indicating that a change in lung volume preceded changes in RRI. These results suggested that the increase in TV activated the pulmonary stretch receptors that contribute to RSA and thus enhanced the baroreflex, resulting in increases in RSA, RSA-TF, and sBRS. The increase in TV activates pulmonary stretch receptors through expanding the lungs, which might enhance the inhibitory effect of vagal nerve activity upon inspiration, which in turn would enhance RSA [41]. Activated pulmonary stretch receptors might reduce chemoreflex sensitivity and consequently enhance the vagal baroreflex, with additional enhancement of cardiovagal baroreflex sensitivity [8–10]. In addition, arterial baroreceptors activated by changes in blood pressure due to respiration also contribute to generating RSA, meaning that the baroreflex is one origin of RSA [41, 42]. Therefore, an enhanced baroreflex might have further increased RSA in the present study.

Although RSA and sBRS increased at 45°, TV might also increase at 45°, if these values increased due to the reasons described above.

### Influence of upper torso flexion on blood volume

The distribution of blood volume generally shifts to the lower extremities according to orthostatic stress and stroke volume decreases as noted above. As a result, sympathetic activity increases, heart rate increases, and vagal activity decreases, that is, cardiovagal baroreflex sensitivity and respiratory sinus arrhythmia decrease [1–6, 43]. However, RSA and sBRS increased during upper torso flexion at 45° and 60° compared with 30° and RSA-TF increased at 60° compared with 30°; this differed from previous findings [1–5, 43]. We preliminarily compared stroke volume and cardiac output using impedance cardiography at upper torso flexion angles of 30° and 60° in five male participants. Stroke volume and cardiac output did not differ between the two conditions (stroke volume: 30° and 60°: 89.52 ± 3.17 and 90.64 ± 3.68 mL; cardiac output: 30° and 60°: 6.12 ± 0.31 and 6.09 ± 0.25 L/min; stroke volume increased in three participants at 30° and cardiac output increased in two at 30°), meaning that gravity at the two tilt angles had little or no influence on blood volume. Thus, blood volume probably did not shift to the lower body and sympathetic activity likely did not change due to increasing upper torso flexion in Fowler’s position. However, these preliminary findings indicate that further studies are required.

## Limitations and conclusions

We studied the influence of slight differences in upper torso flexion on cardiovascular functions and regulation. Our findings indicated the importance of controlling the posture of the upper torso when evaluating the effects of clinical or experimental therapies using measurements of cardiovascular autonomic function based on heart rate and blood pressure variability. We also found that the distribution of blood volume was apparently maintained under upper torso flexion in Fowler’s position with enhanced vagal nerve activity, not sympathetic nerve activity. Moreover, breathing was facilitated, which might benefit patients.

The present findings were generated under conditions of controlled respiration and thus the results should be compared with events under spontaneous breathing. We assessed hemodynamics and respiration at slight angular differences, and in consideration of the influence of fatigue on the participants, we did not assess tidal volume at 45° in Experiment 2. The relationship between hemodynamics and tidal volume at 45° should be assessed.

We found essentially the same tendencies between male and female participants in Experiment 1, so we considered that gender did not affect the present findings. In addition, all our study participants were young. Fowler’s position is used for patients of all ages and we plan to study older individuals in the future. Also, considering that Fowler’s position is used in clinical practice, we also plan to compare Fowler’s and supine positions. Which degree of upper torso incline is optimal for patients under clinical conditions remains unknown. We plan to investigate combinations of various angles in the future. In conclusion, we found that slight differences in upper torso flexion in Fowler’s position influence autonomic cardiovascular regulation and might enhance the baroreflex and contribute to vagal nerve activity in young adults under conditions of a fixed respiratory rate.
